# Knockdown of Selenocysteine-Specific Elongation Factor in *Amblyomma maculatum* Alters the Pathogen Burden of *Rickettsia parkeri* with Epigenetic Control by the Sin3 Histone Deacetylase Corepressor Complex

**DOI:** 10.1371/journal.pone.0082012

**Published:** 2013-11-25

**Authors:** Steven W. Adamson, Rebecca E. Browning, Khemraj Budachetri, José M. C. Ribeiro, Shahid Karim

**Affiliations:** 1 Department of Biological Sciences, the University of Southern Mississippi, Hattiesburg, Mississippi, United States of America; 2 Vector Biology Section, Laboratory of Malaria and Vector Research, National Institute of Allergy and Infectious Diseases (NIAID), National Institutes of Health (NIH), Bethesda, Maryland, United States of America; University of Minnesota, United States of America

## Abstract

Selenocysteine is the 21st naturally-occurring amino acid. Selenoproteins have diverse functions and many remain uncharacterized, but they are typically associated with antioxidant activity. The incorporation of selenocysteine into the nascent polypeptide chain recodes the TGA stop codon and this process depends upon a number of essential factors including the selenocysteine elongation factor (SEF). The transcriptional expression of SEF did not change significantly in tick midguts throughout the blood meal, but decreased in salivary glands to 20% at the end of the fast feeding phase. Since selenoprotein translation requires this specialized elongation factor, we targeted this gene for knockdown by RNAi to gain a global view of the role selenoproteins play in tick physiology. We found no significant differences in tick engorgement and embryogenesis but detected no antioxidant capacity in tick saliva. The transcriptional profile of selenoproteins in *R. parkeri*-infected *Amblyomma maculatum* revealed declined activity of selenoprotein M and catalase and increased activity of selenoprotein O, selenoprotein S, and selenoprotein T. Furthermore, the pathogen burden was significantly altered in SEF-knockdowns. We then determined the global impact of SEF-knockdown by RNA-seq, and mapped huge shifts in secretory gene expression that could be the result of downregulation of the Sin3 histone deacetylase corepressor complex.

## Introduction

The twenty-first amino acid, Selenocysteine (Sec), is incorporated into selenoproteins at the opal (UGA) stop codon. This complex recoding process requires a Selenocysteine-incorporation sequence element (SECIS) in the 3’-UTR of all eukaryotic selenoprotein mRNAs except the Selenoprotein N, which is able to support UGA read-through in the absence of a SECIS element based on the presence its own unique stem-loop sequence within the coding region [1]. Additionally, this process of co-translational insertion of Sec requires a SECIS binding protein 2, ribosomal protein L30, and a Sec-specific translation elongation factor (SEF) that specifically binds to the Sec-tRNA^[Ser]Sec^ [2-5]. 

Selenoproteins play critical roles in the reduction of reactive oxygen species produced by mitochondrial oxidative phosphorylation, NADH/NADPH oxidase, P-450 monooxygenase, lipoxygenase, cyclooxygenase, xanthine oxidase, etc. [6]. Surprisingly, higher plants, fungi and at least five insect species *do not* contain selenoproteins: *Tribolium castaneum, Bombyx mori, Drosophila willistoni, Apis mellifera* and *Nasonia vitripennis* [7-9]. Instead, they possess cysteine-containing homologs or may lack selenoproteins altogether, and certainly where they are present, the selenoproteome seems to be reduced to 1-3 selenoproteins, such as in *Drosophila melanogaster* and *Anopheles gambiae* [10]. The evolutionary reduction in the use of selenoproteins may be linked to significant changes in insect antioxidant defense systems [11-13]. 

The tick genome encodes a number of antioxidants that combat the host defense system and counteract the reactive oxygen species produced during the digestion of heme and as a byproduct of normal cellular processes [14]. Although tick selenoproteins have been scarcely investigated, there is evidence to suggest they may also play critical roles at the vector-pathogen-host interface. Glutathione peroxidase (GPx/Salp25d) in *Ixodes scapularis* saliva plays its well-characterized role in the peroxide detoxification but was also found to be important in the acquisition of *Borrelia burgdorferi* spirochetes from murine hosts [15]. Sep15/SelM associates with the UDP-glucose: glycoprotein glucosyltransferase (UGTR), a complex responsible for maintaining proper protein folding in the endoplasmic reticulum and one study has shown that the expression of SelM is upregulated in *Dermacenter variabilis* infected with *Anaplasma marginale*, and infection levels in tick guts were reduced after SelM knockdown [16]. In the salivary glands of the hard tick *Hyalomma asiaticum asiaticum*, Hyalomin–A and –B were found to suppress host inflammatory responses by modulating cytokine secretion and detoxifying reactive oxygen species [17]. 

In this study, we have examined the impact of selenoproteins at the organismal level, by targeting the selenocysteine-specific elongation factor, an essential factor in selenoprotein biosynthesis, for depletion using RNA interference [18,19]. We examined the effect this knockdown had on the transcriptional expression of selenogenes in the Gulf-coast tick, *Amblyomma maculatum*, that were pathogen-free or infected with *Rickettsia parkeri*, which causes a mild febrile illness similar to Rocky Mountain spotted fever [20,21]. It is apparent that selenoproteins are not essential to feeding, vitellogenesis or fecundity, but significantly impacted salivary gland total antioxidant capacity. It is noteworthy that the transcriptional levels of some selenogenes were significantly lower in pathogen-infected ticks and that the pathogen burden and distribution was altered in the SEF knockdowns. 

## Results

### Bioinformatic analyses

During the course of a massive EST sequencing project identifying transcripts in *A. maculatum* salivary glands [22], we identified an open reading frame with significant amino acid homology to arthropod SEF sequences. The AmSEF amino acid sequence (GenBank ID: AGP03156) contains a GTP/Mg2+ binding site, guanine nucleotide exchange factor interaction site, Switch I and II regions, and G1-5 box regulatory sites [23]. A search in the conserved domain database indicated the presence of the SelB_euk (cd01889) and SelB_II (cd03696) domains with predicted E-values of 6.31e^-70^ and 2.13e^-35^, respectively [23]. These domains take their name from the bacterial selenocysteine-specific elongation factor, which is encoded by the SelB gene. In bacteria, the C-terminal part of SelB recognizes the SECIS hairpin structure, while the N-terminal region binds GTP and tRNA in analogy with elongation factor Tu (EF-Tu) [24]. Although archaeal and eukaryotic mechanisms of selenocysteine incorporation are more complex, they both require a specific selenocysteine-specific elongation factor used during the recoding process. 

**Figure 1:**
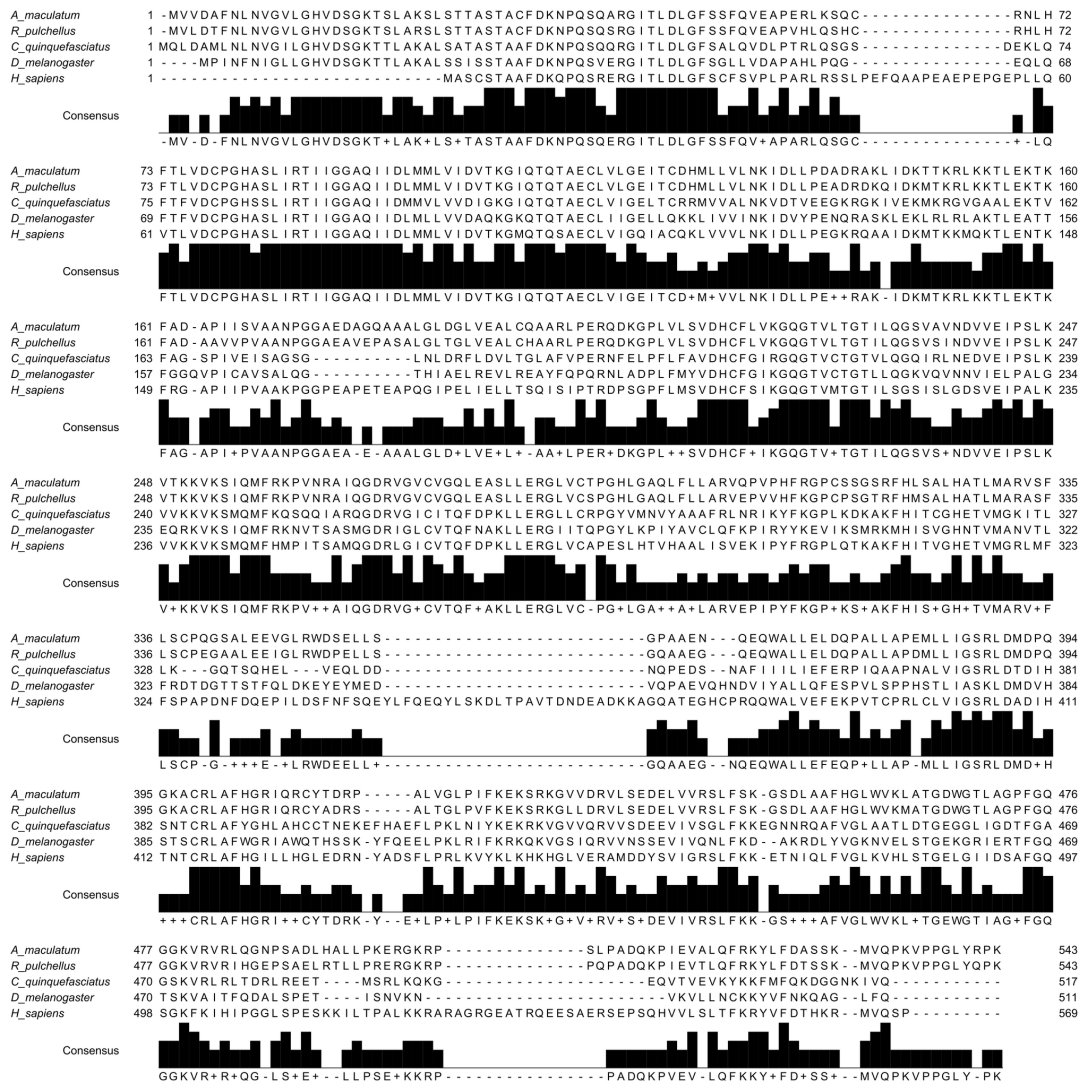
Multiple sequence alignment. Multiple sequence alignment of the SEF amino acid sequence from *Amblyomma maculatum, Rhipicephalus pulchellus, Culex quinquefasciatus, Drosophila melanogaster*, and *Homo sapiens*.

Eighty-nine percent amino acid identity was found between the SEF amino acid sequences of *A. maculatum* and the zebra tick, *Rhipicephalus pulchellus* (Figure 1). SEF orthologs from *Culex quinquefasciatus*, *Drosophila melanogaster*, and *Homo sapiens* had amino acid similarity between 50-59% (44-55% identity) when compared to the AmSEF sequence. SEF sequences were absent from many of the model invertebrate species, which is consistent with the apparent rarity of selenoproteins in many insect species observed.

**Figure 2:**
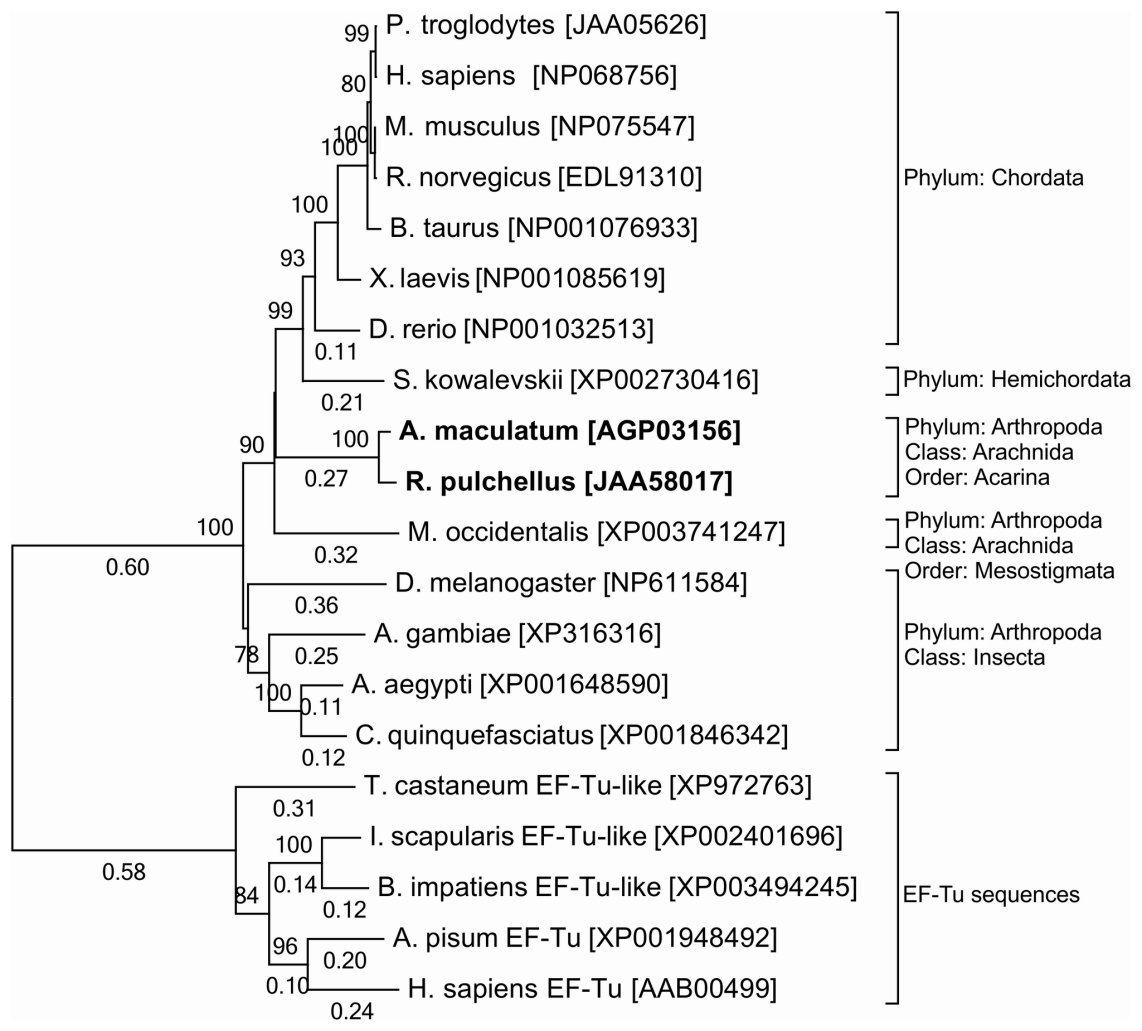
Evolutionary relationships of taxa based on the SEF amino acid sequence using the Neighbor-joining method. Bootstrap values <70 and branch lengths shorter than 0.10 were not displayed.

A phylogenetic tree was constructed using the AmSEF amino acid sequence and orthologs from diverse genera and was found to be congruent with traditional taxonomic classifications, indicating that SEF behaves like an orthologous family (Figure 2). Only a single SEF gene was found in any examined species. The tick SEF proteins group within the arthropods as expected and were related to three other predatory or blood-sucking arthropods, *Metaseiulus occidentalis*, *C. quinquefasciatus, Aedes aegypti*, *Anopheles gambiae*, as well as *D. melanogaster* (Figure 2), further confirming their identity as SEF orthologs. 

The sequenced genome of *I. scapularis* contains a SEF sequence which likely encodes for elongation factor Tu [14] (Figure 2) , which has been proposed to be the ancestral gene of SEF [24-26]. Sequences of elongation factor Tu from *I. scapularis, Acrythosiphon pisum, Tribolium castaneum, Bombus impatiens* and *H. sapiens* are an outgroup on the phylogenetic tree (Figure 2). It is possible that *I. scapularis* lacks this essential component of selenocysteine incorporation, and selenocysteine incorporation could be limited to the metastriate lineage (including *A. maculatum* and *R. pulchellus*). The biological distinctions between prostriates (including *I. scapularis*), and metastriates include differences in the type of developmental cycle (three-host vs. one-host), host range and vector competence has a genetic basis [27] that may include differences in selenoprotein incorporation. Alternatively, it is possible that the gene encoding SEF in *I. scapularis* is present but has not yet been annotated. 

### Transcriptional gene expression

**Figure 3:**
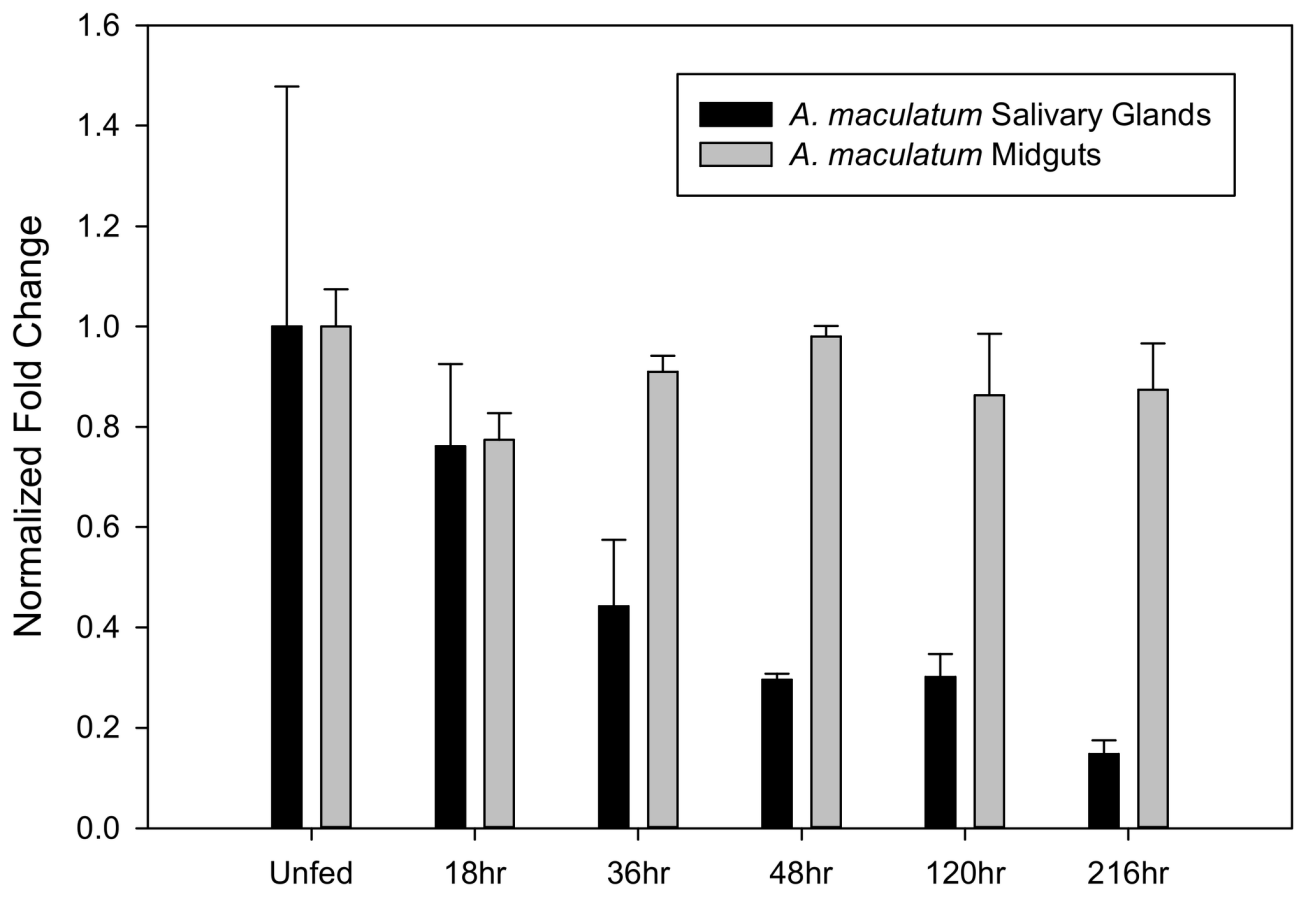
Transcriptional expression of SEF in tick tissues throughout the blood meal. Normalized fold change in the transcriptional activity of SEF in *A. maculatum* salivary glands and midgut tissues throughout the blood meal.

 The relative transcriptional expression of SEF was studied in order to gain a better perspective on the role of SEF with respect to functions related to blood feeding and its contribution to selenoprotein expression. Within salivary glands, the *sef* transcript levels were highest in the unfed tick, and gradually declined ~80% in the repleted tick (Figure 3). In tick midguts, transcriptional expression appeared to be fairly consistent over the blood meal. These data suggest that the transcriptional control is principally operating to maintain relatively consistent levels of the *sef* transcript, particularly within midguts. Even within salivary glands, the degree of variance is fairly small over the course of the blood meal. The slow decline in salivary gland *sef* transcript could potentially be linked to global genetic shifts towards oviposition and senescence. In *Drosophila*, the *sef* transcripts were abundantly expressed at high levels in the embryo and pupa and slowly declined throughout development [28]; although *Drosophila* and ticks have a different developmental program, there are clear similarities between the two.

### RNA interference

**Figure 4:**
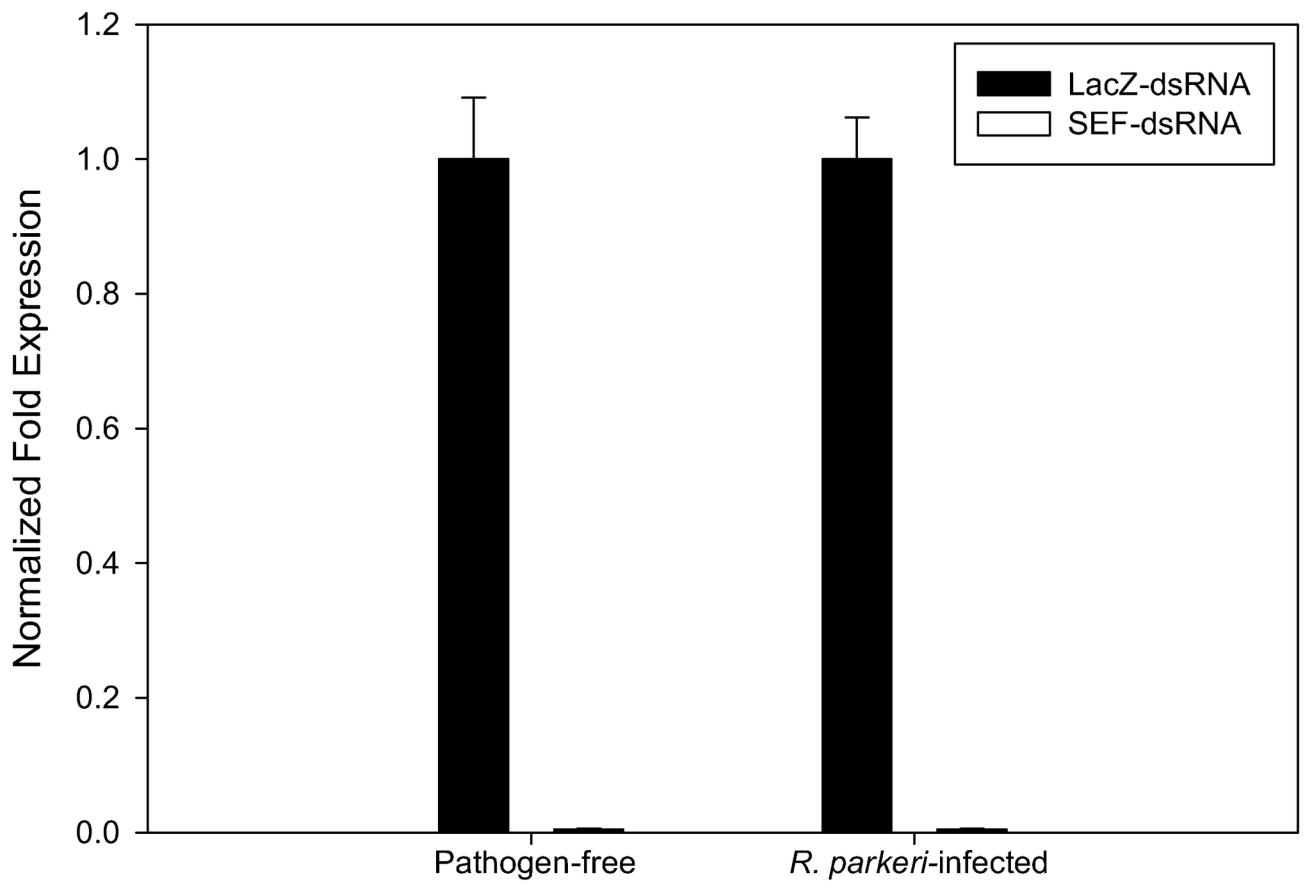
Evidence of transcriptional knockdown of SEF in dsRNA-injected ticks. Normalized fold change in the salivary gland transcriptional activity of SEF in pathogen-free and *R. parkeri*-infected *A. maculatum* injected with LacZ-dsRNA or SEF-dsRNA.

**Figure 5:**
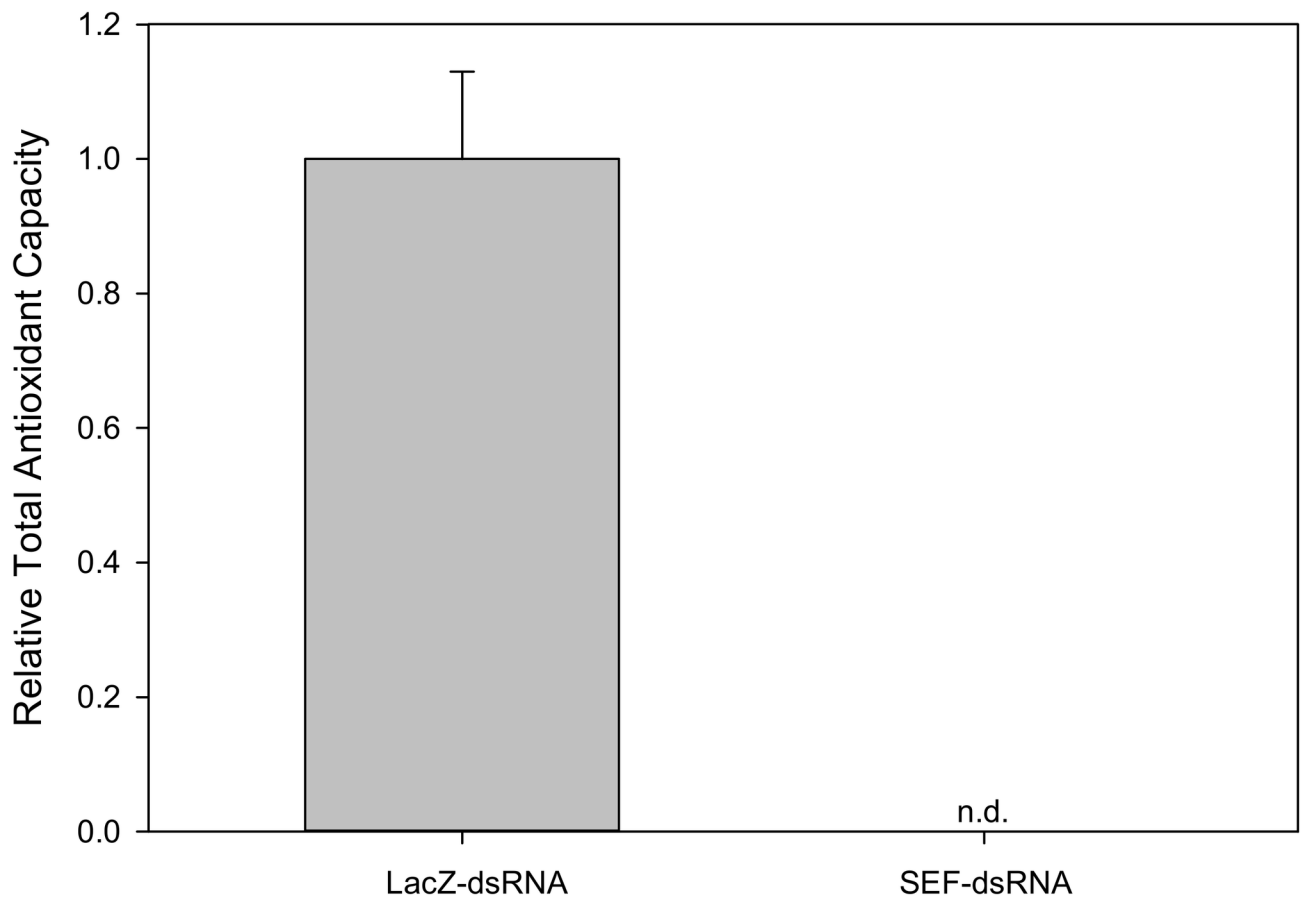
Total antioxidant capacity. Relative total antioxidant capacity in pooled saliva collected seven days post-infestation from pathogen-free *A. maculatum* injected with LacZ-dsRNA or SEF-dsRNA. (n.d.: not detected).

 In order to understand how selenoproteins affect the interactions between the vector and pathogen, we induced RNAi-mediated knockdown of the *sef* gene in *A. maculatum* that were either naturally infected with *R. parkeri* or were determined to be free of any Rickettsial pathogen. QRT-PCR analysis shows a >99.5% decrease in transcripts in ticks injected with SEF-dsRNA at 5-days post-infestation (Figure 4). We then assessed the effect of *sef* gene knockdown on the average engorged body weight, egg mass, egg conversion ratio (egg mass/tick body weight) and hatchability and noted no significant differences from controls in any tested variables (Table 1). This is congruent with the observations in *Drosophila* sef knockout mutants which were viable, fertile and had the same mean lifespan as controls [28]. We then assayed tick saliva (collected at 7 days post-infestation) for the total antioxidant capacity from control and *sef* knockdowns and, surprisingly, we did not detect any activity in tick saliva in the *sef* knockdowns (Figure 5). A previous report has shown that selenoproteins were not essential for redox homeostasis in *D. melanogaster* since control and *sef*-knockout mutants had similar survival rates when exposed to H_2_O_2_ and paraquat [28]. It is thought that this may result from the activity of cysteine analogs of several enzymes which are principally involved in reactive oxygen reactions, such as glutathione peroxidase, thioredoxin reductase, methionine sulfoxide reductase, etc. [13,29,30]. Based on sequence analysis, it is evident the tick sequences of these key antioxidant enzymes have not lost their selenocysteine coding capacity, at least in *A. maculatum*, which may explain the apparent inconsistency between these two data sets.

**Table 1:** Phenotypic comparison of ticks injected with LacZ-dsRNA or SEF-dsRNA.

**Test Group**	**# of Ticks**	**Mean Body Weight (mg)**	**Mean Egg Mass (mg)**	**Egg Conversion Ratio**	**Hatchability**
Pathogen-free LacZ-dsRNA	3	515.3 ± 128.9	258.6 ± 101.5	0.4651 ± 0.073	++++
Pathogen-free SEF-dsRNA	9	264.9 ± 34.9	94.5 ± 21.0	0.3168 ± 0.042	++++
*R. parkeri*-infected LacZ-dsRNA	7	796.1 ± 137.4	373.9 ± 92.0	0.4105 ± 0.063	++++
*R. parkeri*-infected SEF-dsRNA	13	602.1 ± 84.9	316.0 ± 65.0	0.4432 ± 0.061	++++

**Figure 6:**
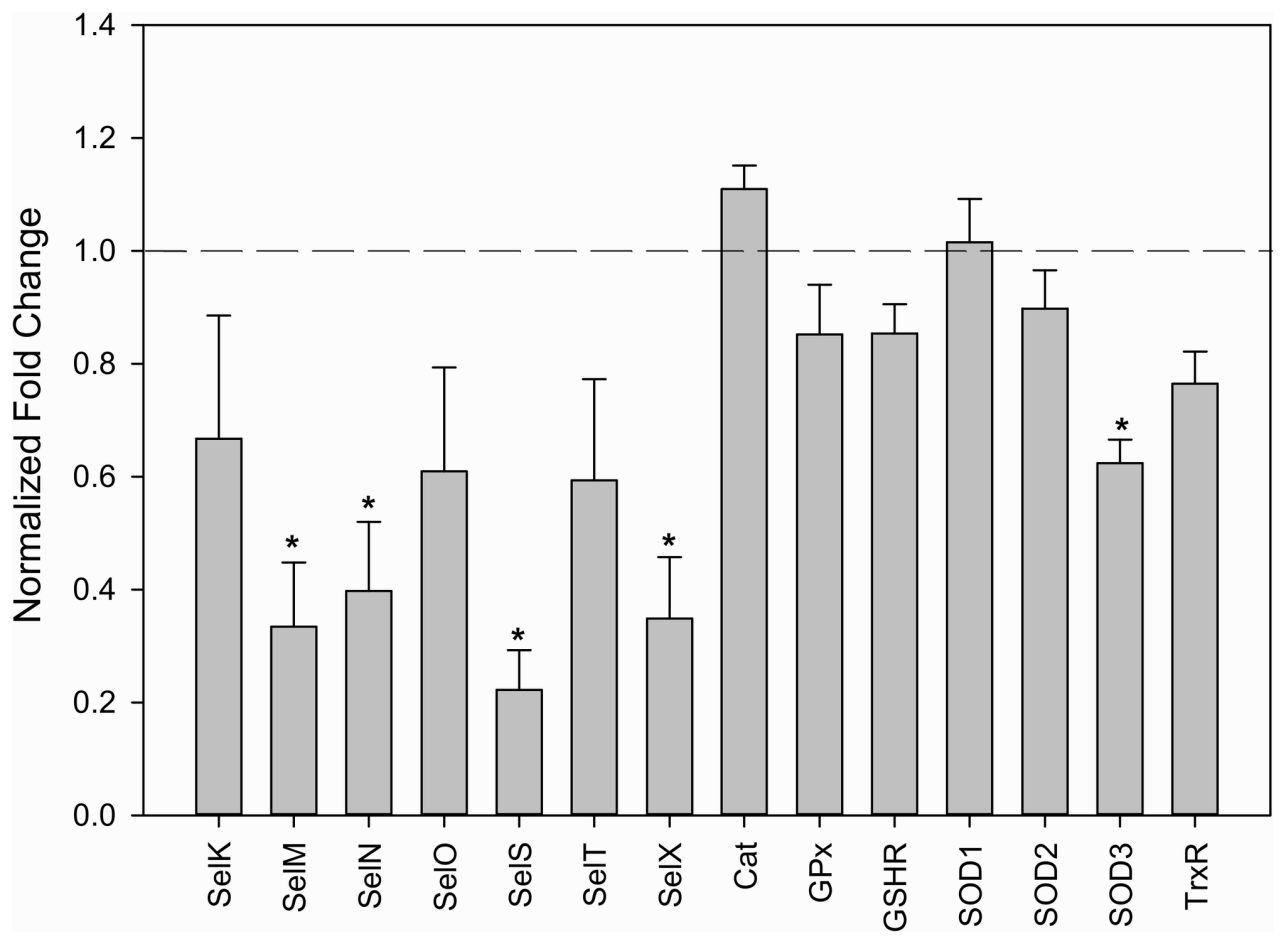
Effects of SEF-knockdown on selenogenes and antioxidant genes. Normalized fold change in the transcriptional activity of selected selenogenes and associated antioxidant genes in the salivary glands of pathogen-free *A. maculatum* blood fed seven days and preceded by SEF-dsRNA injection. The transcriptional level of each candidate gene in tissues injected with LacZ-dsRNA was set to 1.0 as a reference point.

We then examined the transcriptional levels of all available selenoprotein genes identified in the *A. maculatum* sialotranscriptome [22] in both the control and the *sef* knockdown and noted a general decrease in the transcriptional expression of all tested selenoprotein genes and a statistically significant decrease of *selM, selN, selS*, and *selX* at 5-days post-infestation although all were less than 5-fold downregulated (Figure 6). This could suggest that at the organismal level, the expression of selenoproteins may have been downregulated due to insufficient selenocysteine incorporation machinery and/or activation of complementary systems. The cellular ratio of selenoprotein translational machinery is an essential determinant for expression, at least in bacterial selenoprotein synthesis [31]. In bacteria, the decoding of the opal stop codon requires the formation of a quaternary complex between the bacterial selenocysteine-specific elongation factor termed “SelB”, GTP, selenocysteyl-tRNA^Sec^ and the SECIS element, which is greatly favored with a balanced component ratio [26,31]. 

**Table 2:** Sequences of oligonucleotide PCR primers used in this study.

**Gene**	**GenBank ID**	**Forward primer (5’-3’)**	**Reverse primer (5’-3’)**	**Size (bp)**
Act	JO842238	TGGCTCCTTCCACCATGAAGATCA	TAGAAGCACTTGCGGTGCACAATG	169
Cat	JO843741	AAAGGACGTCGACATGTTCTGGGA	ACTTGCAGTAGACTGCCTCGTTGT	173
GPx	JO843645	TGCCGCGCTGTCTTTATTATTGGC	AGTTGCACGGAGAACCTCATCGAA	102
GSHR	JO844062	ACCTGACCAAGAGCAACGTTGAGA	ATCGCTTGTGATGCCAAACTCTGC	170
OmpB	AF123717	CAAATGTTGCAGTTCCTCTAAATG	AAAACAAACCGTTAAAACTACCG	96
		Probe: 6-FAM-CGCGAAATTAATACCCTTATGAGCAGCAGTCGCG-BHQ-1		NA
SEF	KC989559	TGGCTCCAGAAATGCTGCTCATTG	ACGCCTTTGCGACTCTTCTCCTTA	157
SelK	JO843326	AGTTCCAGCAGGTCATCAGTGTCA	TCCAGGAATAGGGCAGTCCATTGT	132
SelM	JO842653	ATGATACCTGAATGGCCATCCGCA	TGATCGCGGGTCATCTTCTCCAAA	171
SelN	KC989560	TTAGTTTGGACACTGTGGACGGGT	AGGCTTCTCTAACAACGGCACTCA	150
SelO	KC989561	AAGCTCGGCCTTGTGAAGAGAGAA	TACAGCACGACAAGAGCTTGGACA	190
SelS	JO842687	AGAACAAGTGCACCACAACAGCAG	ATTTCTTGCATCCTTCGACGTGCC	107
SelT	KC989562	TCTTTGTGTGTGGAGCCATCGAGA	ACCACACCCGCACGTCATTAAAGT	81
SelX	JO845128	ACCACTCTCCTTGGCCATCATTCA	TGCACTTCCCACAGTACACCTTGA	108
SOD1	JO844140	GGAACCGAAGACAGCAAGAA	GAGAAGAGGCCGATGACAAA	143
SOD2	JO843189	AGGCTGTCTGTGTGTTGAAG	TTGCCGAGGCCCTTTATTT	112
SOD3	JO843979	GCATCTACTGGACAAACCTCTC	GCAGACATCAGGCCTTTGA	115
TrxR	JO843723	TGTGACTACACCAACGTGCCTACA	AGTAGCCTGCATCCGTTCCTCTTT	175
Am56960	NA	GTCGTCTTACGGAGAAAGTCTG	GTACACTGGGCTTTGCTTTATTT	104
Am56979	NA	TCGTCGCAACTGCTGATATT	GTATGCCTTCCCGTATCCTTTC	101

The transcriptional expression of four non-selenoprotein enzymes playing crucial roles in ameliorating oxidative stress, catalase (1 gene) and superoxide dismutase (3 genes), were also evaluated in SEF-knockdowns. Although they are not selenoproteins, these enzymes are involved in the remediation of the most damaging free radical, superoxide. Since much cellular damage is caused by the superoxide radical, it is possible that the transcriptional or enzymatic activity might be affected in the SEF knockdowns, particularly since there was a trend toward generally declining transcriptional activity of selenoprotein genes in the SEF knockdowns. In fact, with respect to the control, the transcriptional activity of these genes, only one of the superoxide dismutase genes (SOD3) was significantly lower, and changes in transcriptional expression of SOD1/2 and catalase were not statistically different from controls in the SEF-knockdowns (Figure 6). 

### Evaluating the transcriptional expression of selenoprotein genes in *R. parkeri*-infected ticks

**Figure 7:**
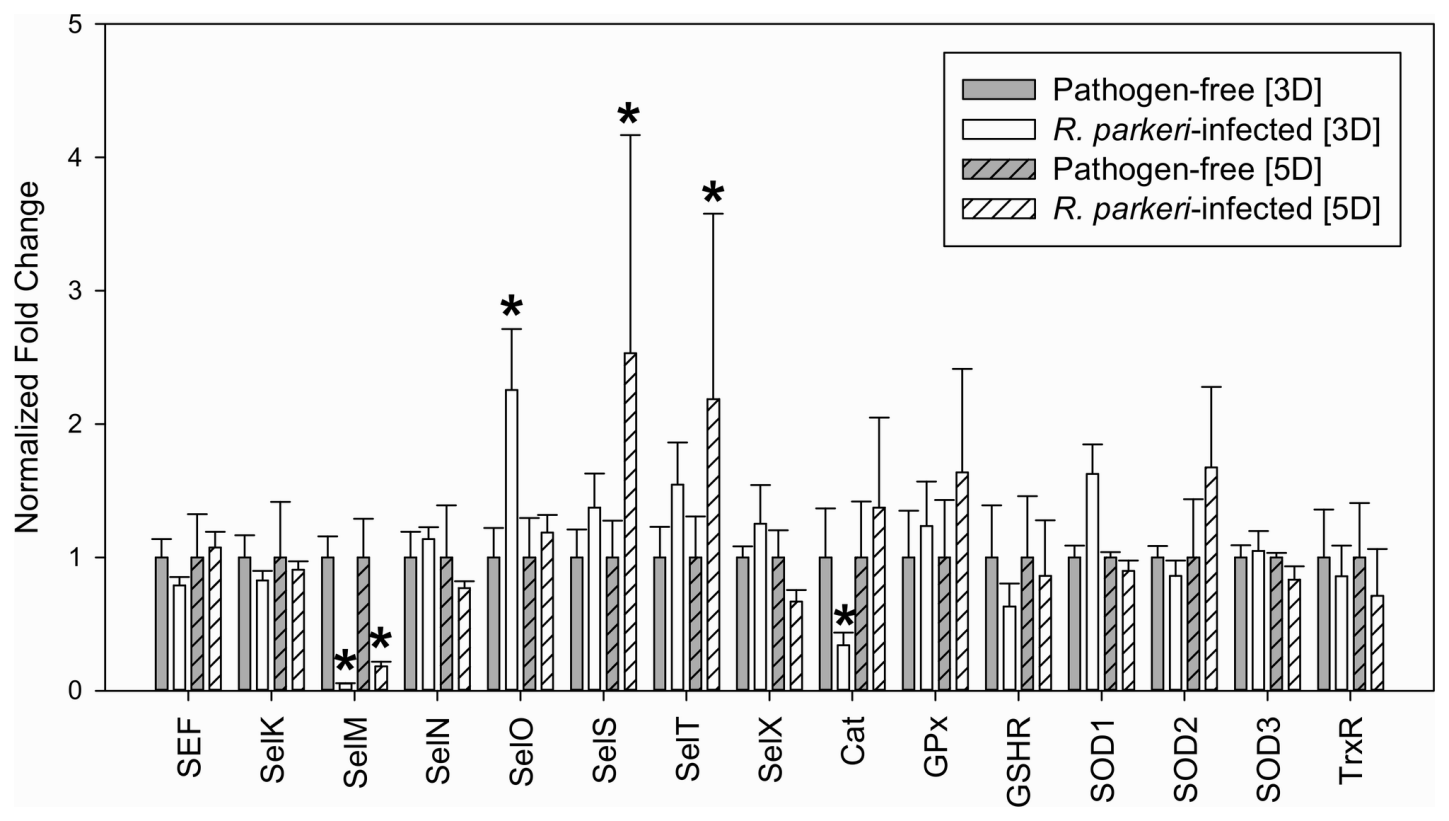
Transcriptional activity of selenogenes in pathogen-free and *R. parkeri-*infected tick midgut tissues. Normalized fold change in the transcriptional activity of selected selenoprotein and associated antioxidant genes in pathogen-free or *R. parkeri-*infected *A. maculatum* midguts at three and five days post-infestation. Gene expression was normalized to pathogen-free levels in three and five days to highlight differences observed in *R. parkeri-*infected tick midguts. (*p<0.05).

 To further understand the role of selenoproteins/antioxidants in pathogen infection, the midgut transcriptional gene expression of selenogenes was examined in pathogen-free ticks and wild-caught *A. maculatum* that were naturally infected with *R. parkeri*. These data show that the transcriptional expression of selenoproteins as a whole remains largely unchanged in the *R. parkeri*-infected SEF-knockdown ticks, however, SelM had significantly lower transcriptional activity in the midguts of infected ticks at both three and five days post-infestation (Figure 7). This is surprising, since the expression of SelM was upregulated in the IDE8 tick cell line infected with the intracellular pathogen, *A. marginale* [32].

SelO had higher transcriptional levels in the infected ticks at three days post-infestation, whereas the same was true for SelS and SelT at five days post-infestation (Figure 7). The function of SelO is currently unknown, but it is suggested to be a putative kinase involved in redox reactions and is widely distributed [33,34], and therefore it is difficult to speculate what its involvement is, if any, in vector-pathogen interactions. SelS is ER-localized and is important for the removal of misfolded proteins from the ER membrane [35]. SelT is ER-localized and likely contains a thioredoxin-like fold based on sequence similarity [6]. The expression of SelT may be involved in stress-related phenomena, including calcium regulation and growth hormone secretion [36].

Additionally, the transcriptional expression of catalase is significantly lower in the *R. parkeri*-infected midgut at three days post-infestation. It is noteworthy that one of the dominant microbial control systems in *D. melanogaster*, maintains the redox balance of the fly gut and has critical importance to the innate immune system [37,38]. This two-component system includes a dual oxidase that generates ROS to oxidize and kill bacteria and an immune-regulated extracellular catalase that removes excess luminal ROS that might otherwise harm the gut epithelium [37,38]. This system seems to be partially operative in another blood-feeding arthropod, *A. aegypti* [39]. It is possible that the decrease in transcriptional activity of catalase in the SEF-knockdown is modulated by *R. parkeri* in order to evade oxidative killing. Alternatively, it may be a tick response to pathogens serving to increase the amounts of H_2_O_2_ available for the formation of peroxynitrite [40]. 

There is increasing evidence to suggest that redox signals affect vector-pathogen interactions, including pathogen entry through protein redox switches and redox modification [41,42]. In some cases, the innate immune system combats pathogen infection by activation of a primitive apoptotic cascade [43]. Moreover, ER stress originating from the innate immune response leads to an unfolded protein response or apoptosis when ER function cannot be restored [44]. Taken collaboratively, this data set suggests that the successful dissemination of *R. parkeri* might require the management of ER stress by modulating selenoprotein factors (SelS/SelM) which participate in protein folding/disulfide bond formation. 

### Analysis of *R. parkeri* pathogen burden in SEF knockdown ticks

**Figure 8:**
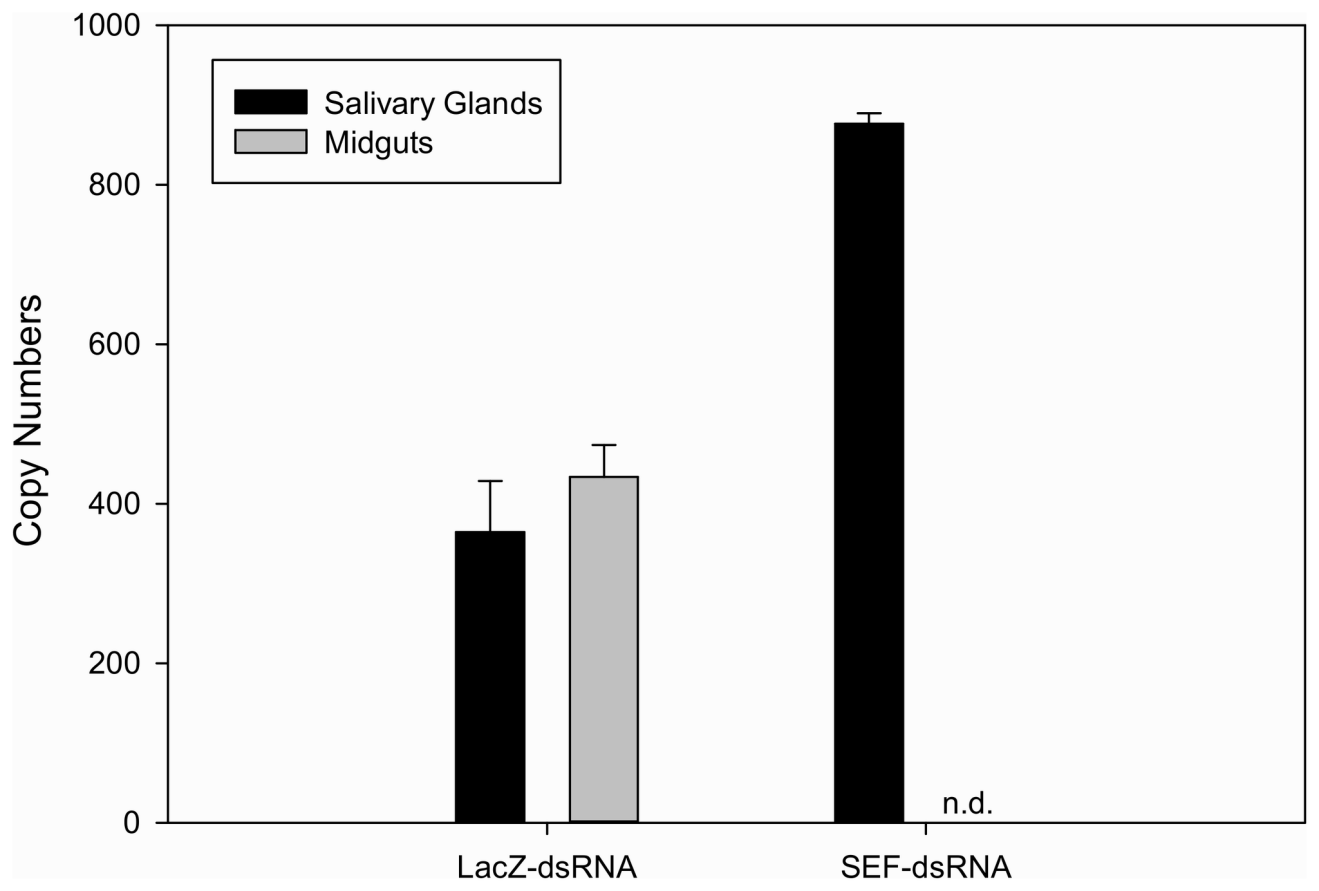
*R. parkeri* pathogen burden in RNAi tissues. (n.d.: not detected).

 Since it is clear that *R. parkeri* infection influences the transcriptional activity of some tick selenogenes, we then determined the pathogen burden within *R. parkeri*-infected ticks injected with control or SEF-dsRNA using a probe-based QRT-PCR assay using cDNA prepared from *A. maculatum* tissues. These data reveal that the total number of live *R. parkeri* is approximately equivalent in both control and SEF knockdown ticks, however, there was no detectable *R. parkeri* in the midguts of the SEF-knockdown ticks (evaluated at 7-day post-infestation) (Figure 8). In a previous experiment [16] where the SelM transcript was silenced, there was almost no bacteria in the salivary glands of *A. marginale*-infected *D. variabilis*, and the pathogen load in both midguts and salivary glands was considerably lower than controls. This may be a reflection of the fact that artificial feeding by capillary tube was used to inoculate *D. variabilis* with *A. marginale* [16], whereas in this current study *R. parkeri*-infected *A. maculatum* were naturally infected and had maintained this rickettsial infection through transovarial transmission or by acquisition of an infected blood meal. It is noteworthy, that the previous study used DNA template for pathogen detection, so it is not possible to gauge the number of living bacteria, which may have also contributed to the observed differences in these two studies. It is possible that lower levels of catalase in the *R. parkeri*-infected *A. maculatum* midgut tissues could have resulted in excess luminal ROS, damaging the epithelium, and creating an environment which was conducive to the colonization of *R. parkeri*, but this was not affected in salivary glands.

### Impact of SEF silencing on the sialome of *A. maculatum*

 Based on the observed relationship between selenoproteins and *R. parkeri*, we sought to determine the global changes in gene expression of *R. parkeri*-infected *A. maculatum* salivary glands. RNA-seq data was obtained from ticks naturally infected with *R. parkeri* injected with SEF-dsRNA and also from controls injected with LacZ-dsRNA. These reads were used to reassemble and extend a sialotranscriptome of *A. maculatum* from which 25,024 putative coding sequences (CDS) were extracted (file S1). A total of 43,161,335 and 45,085,589 reads were mapped back to these CDS from the SEF and LacZ libraries, respectively. For each contig, the number of mapped reads from each library was compared by a Χ^2^ test, and the normalized ratio from their reads was computed (with the denominator added to 1 to avoid division by zero). By sorting the data by significance and the ratios, the differences between the two sets become apparent. 

First, we observed that 359 CDS became significantly reduced 5-fold or more with SEF silencing. The class of putative secreted proteins was down regulated 55%, exhibiting twice the rate of change in expression for any other category, most likely as a result of the secretory nature of salivary gland physiology. Other classes such as signal transduction, unknown conserved, protein synthesis machinery and protein export machinery were significantly underrepresented in the 5-fold downregulated SEF group (Table 3). The SEF transcript itself was downregulated 5-fold (80%, 4 reads in the SEF library against 26 in the control), but at the limit of detection due to low expression. Selenoprotein M (Am-13529, file S1) was found 9.75-fold suppressed, and selenoprotein T was suppressed 4.3-fold (Ambmac-359). When looking at the extremely downregulated CDS, those with 100-fold or more decreased expression upon SEF silencing, we observe in this group of 45 CDS that 33 belong to the secreted class, nine to the unknown class, one is involved in lipid metabolism and one in nuclear regulation. Among the CDS of the secreted class, Ambmac-66586 has 13,121 mapped reads of the control library and zero from the SEF suppressed. An acyl-CoA synthase is over 100-fold suppressed in the SEF library. If we normalize the results between libraries by the same actin transcript used to normalize the qPCR experiment reported in Figure 6, we obtain similar results between RNA-seq and qPCR experiments. Because the actin transcript is relatively more expressed (4.71-fold) in the SEF dsRNA-injected library, the relative reduction of the SEF transcript which was 5-fold as indicated above becomes 23.5-fold when normalized to the actin transcript (Table 4).

**Table 3:** RNA-seq of SEF vs LacZ-dsRNA injected ticks.

**Class**	**Total # of Contigs**	**% Total**	**# of contigs decreased ≥5-fold following RNAi**	**%**	**% Down / % Total**	***P***	**# of contigs increased ≥5-fold following RNAi**	**%**	**% Up / % total**	***P***
Secreted	6375	25.477	200	55.710	2.186	0.0000	477	54.702	2.147	0.0000
Unknown	6123	24.469	75	20.891	0.854		158	18.119	0.740	0.0002
Protein modification machinery	1081	4.320	11	3.064	0.709		42	4.817	1.115	
Extracellular matrix/cell adhesion	437	1.746	3	0.836	0.477		35	4.014	2.298	0.0000
Unknown, conserved	2045	8.172	6	1.671	0.204	0.0000	28	3.211	0.393	0.0000
Signal transduction	1286	5.139	9	2.507	0.487	0.0007	22	2.523	0.491	0.0007
Cytoskeletal	383	1.531	1	0.279	0.182		18	2.064	1.349	
Metabolism, nucleotide	209	0.835	4	1.114	1.328		14	1.606	1.922	0.0160
Protein synthesis machinery	883	3.529	4	1.114	0.315	0.0002	10	1.147	0.325	0.0002
Transporters/storage	664	2.654	5	1.393	0.524		10	1.147	0.432	0.0067
Oxidant metabolism/detoxification	368	1.471	1	0.279	0.189		9	1.032	0.702	
Transposable element	1181	4.720	15	4.178	0.885		9	1.032	0.219	0.0000
Transcription machinery	979	3.912	7	1.950	0.498		8	0.917	0.234	0.0000
Metabolism, lipid	447	1.786	9	2.507	1.400		7	0.803	0.449	0.0311
Immunity	152	0.607	0				5	0.573	0.944	
Metabolism, energy	342	1.367	0				5	0.573	0.420	0.0467
Protein export machinery	509	2.034	1	0.279	0.137	0.0026	5	0.573	0.282	0.0026
Metabolism, carbohydrate	418	1.670	2	0.557	0.333		4	0.459	0.275	0.0059
Metabolism, amino acid	161	0.643	4	1.114	1.721		3	0.344	0.535	
Nuclear regulation	377	1.507	2	0.557	0.369		1	0.115	0.076	0.0008
Storage	10	0.040	0				1	0.115	2.870	
Transcription factor	195	0.779	0				1	0.115	0.147	0.0266
Nuclear export	37	0.148	0				0	0.000		
Metabolism, intermediate	84	0.336	0				0			
Proteasome machinery	277	1.107	0				0			0.0019
Total	25023	100	359	100			872	100		

Functional classification of transcripts in the whole data set and in those 5-fold or more suppressed following SEF RNAi injection.

**Table 4:** RNA-seq analysis of selenogenes and other antioxidant genes.

**Protein**	**AmSE reads**	**LacZ reads**	**P**	**Significant?**	SEF x k /(LacZ+1) *	**LacZ/[k x (SEF+1))**	**SEF/LacZ normalized by actin**	**LacZ/(SEF+1) normalized by actin**
SelN	7	2	0.0832		2.44	0.24	0.517834	1.127268
TrxR	319	293	0.1117		1.13	0.88	0.2408	4.128617
SelM	762	800	0.9206		0.99	1.00	0.211123	4.727728
SelS	386	409	0.8407		0.98	1.01	0.208938	4.7654
GPX	257	292	0.3256		0.92	1.08	0.194661	5.103289
SelO	18	21	0.7306		0.85	1.06	0.181578	4.983709
SelX	3	8	0.1511		0.35	1.91	0.073976	9.018141
Actin	7953	1764	0.0000	Y	4.71	0.21	1	1
SOD2	674	387	0.0000	Y	1.81	0.55	0.385516	2.5852
GSHR	82	53	0.0060	Y	1.59	0.61	0.337003	2.879286
SOD1	118	85	0.0086	Y	1.43	0.68	0.304507	3.220764
SelT	88	65	0.0332	Y	1.39	0.70	0.295905	3.293141
SelK	681	514	0.0000	Y	1.38	0.72	0.293463	3.398332
SOD3	772	592	0.0000	Y	1.36	0.73	0.288919	3.45326
Catalase	395	307	0.0001	Y	1.34	0.74	0.284617	3.495668
SelT_R	264	214	0.0057	Y	1.28	0.77	0.272508	3.641287
SEF_R	4	26	0.0001	Y	0.15	4.98	0.032878	23.44717

RNA-seq results reporting transcriptional activity of selected selenoproteins and associated antioxidant genes in *A. maculatum* injected with SEF- or LacZ-dsRNA. K is a constant derived from the ratio of total reads between LacZ and SEF dsRNA libraries.

Accordingly, SelN, TrxR, SelM, SelS, GPX, SelO and SelX do not reach statistical significance, some of them due to low read counts, but SOD2, GSHR, SOD1, SelT_R, SelK, SOD3, Catalase and SelT appear significantly reduced in reads between libraries, ranging from 0.38 to 0.27-fold related to the control. These results are similar to those reported in Figure 6, but Figure 6 experiments were done with pathogen-free ticks while Table 4 results originate from naturally-infected ticks.

**Figure 9:**
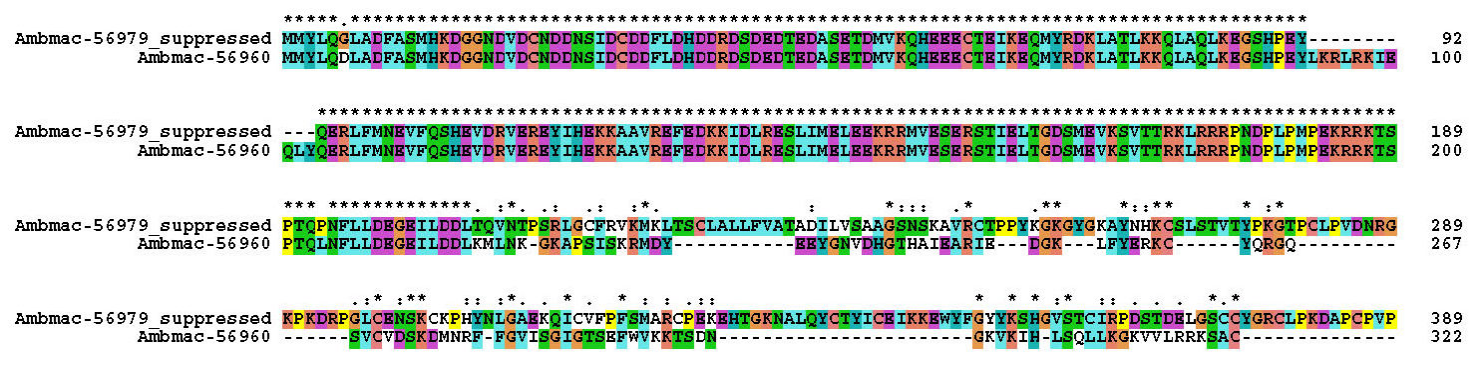
Clustal alignment of two sequences corresponding to SDS3. Clustal alignment of two products coding for component SDS3 of the Sin3 histone deacetylase corepressor complex. Ambmac-56979 appears downregulated following SEF silencing, while Ambmac-56960 does not change with treatment.

**Figure 10:**
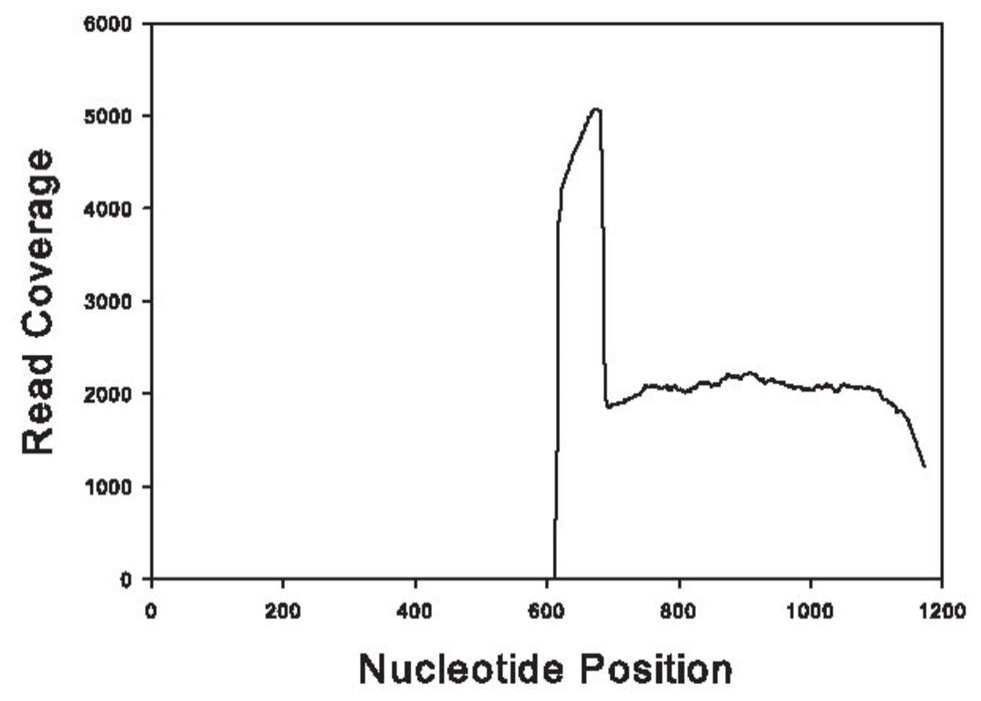
Number of hits per nucleotide site derived from the LacZ control library on Ambmac-56979 transcript.

**Figure 11:**
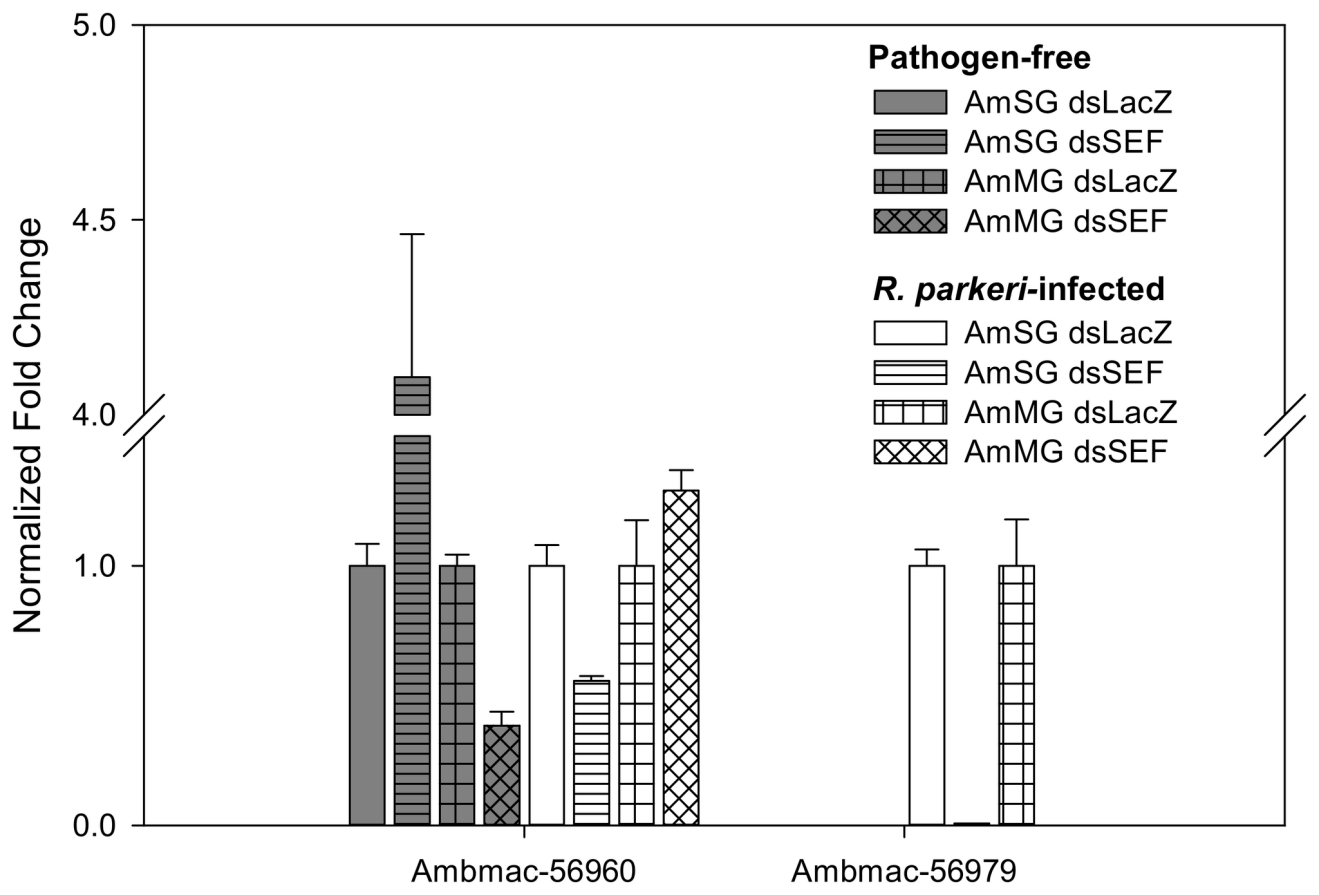
Evidence of transcriptional knockdown of SDS3 components in control and SEF-knockdowns. Transcriptional gene expression of two SDS3 components of the Sin3 histone deacetylase corepressor complexes in LacZ- and SEF-dsRNA knockdowns in pathogen-free and *R. parkeri-*infected *A. maculatum*.

Second, and somewhat surprisingly, a CDS coding for component SDS3 of the Sin3 histone deacetylase corepressor complex (Ambmac-56979) was nearly 3,000-fold suppressed in the SEF library, where only 4 reads were found as opposed to 15,074 in the control. With actin normalization, the difference would be 5 times higher. On closer inspection, another CDS coding for a similar product (Ambmac-56960) was found that matched the first 200 amino acids of Ambmac-56979 (Figure 9), but its expression was not changed with SEF silencing. Inspection of the target sites of the reads on Ambmac-56979 revealed that virtually all reads target the non-homologous region following nucleotide position 600 thus explaining why the reads did not map also to the homologous CDS (Figure 10). This result may derive possibly from misassembly of Ambmac-56979 in a chimera and that the reads deriving from the LacZ library could be targeting something else than component SDS3 of the Sin3 histone deacetylase corepressor complex. Transcriptional gene expression of Ambmac-56960 and Ambmac-56979 was examined in pathogen-free and *R. parkeri*-infected tick tissues. Within the *R. parkeri*-infected tissues, the transcript level of Ambmac-56979 were reduced by 99% and undetectable in salivary glands and midguts, respectively, but this transcript was undetectable in any of the pathogen-free tick tissues (Figure 11). The transcript levels of Ambmac-56960 exhibited differences between the control and the SEF-knockdown, but exhibited different trends in pathogen-free and *R. parkeri*-infected tissues. Ambmac-56960 was upregulated >4-fold in pathogen-free salivary glands compared to control, but down-regulated in *R. parkeri*-infected glands. Conversely, Ambmac-56960 was down regulated in pathogen-free midguts but was not significantly different from control in *R. parkeri*-infected midguts. 

Finally, we found 872 transcripts that are significantly (≥5-fold) upregulated following SEF silencing, the majority of which are also of the secreted (54.7%) and unknown (18.1%) class (Table 3 and file S1). We found only two transcripts associated with oxidant metabolism that were increased, for a peroxidase (Am-4183, 17.7-fold upregulated) and for a cytochrome P450 enzyme (Ambmac-3189, 5.22-fold upregulated). It is not clear whether these transcripts could be increased as a physiological compensation for the reduction in antioxidant products due to SEF suppression. In conclusion, the majority of the affected transcripts were of the secreted class, and this may reflect the differential expression of these transcripts due to the time course or stress that the tick may be subjected to, as their salivary proteins derive mostly from multigenic families that switch expression during feeding, and not due directly to selenoprotein deficiency. However, it is intriguing that the gene expression differences may be attributed to the downregulation of the component SDS3 of the Sin3 histone deacetylase corepressor complex, which should have broad epigenetic repercussions on gene expression [45].

## Discussion

 The purpose of this study was to elucidate the global impact of tick selenoproteins on tick physiology and vector-pathogen interactions. Bioinformatics analyses indicate the amino acid sequence for *A. maculatum* SEF is homologous to other known SEF genes (Figure 1) and includes all of the critical GTP/Mg^2+^ binding sites, guanine nucleotide exchange factor interaction sites, etc., necessary for incorporating the Sec residue into the nascent polypeptide chain. Moreover, the *A. maculatum* SEF sequence is close in phylogenetic lineage to a putative SEF sequence from another metastriate tick species, *R. pulchellus* (Figure 2). Transcriptional activity of SEF remains constant in the midgut tissues throughout the blood meal, whereas in salivary gland tissues, SEF transcripts decline steadily once feeding begins to less than 20% in replete ticks (Figure 3), and may be related to shifts in global transcriptional activities associated with organismal senescence and oviposition. Furthermore, while we achieved >99.5% transcriptional knockdown of SEF (Figure 4), we did not observe any significant differences in engorged body weight, egg mass, egg conversion ratio, and hatchability (Table 1), which is similar to a previous studies demonstrating that *Drosophila sef* knockout mutants were viable, fertile, and had similar mean lifespan as controls [28]. 

In contrast to *A. maculatum*, the *D. melanogaster* genome encodes only three selenogenes (SelH, SelK, and SPS2) [8], and redox homeostasis was not significantly affected in SEF-knockouts [28], whereas in *A. maculatum* no antioxidant capacity was detectable in tick saliva (Figure 5). This is not to suggest that no antioxidant activity was present in ticks, but rather that no antioxidant activity was detectable in tick saliva, particularly since we observed no differences in salivary gland antioxidant capacity between SEF-knockdown and controls (data not shown). This is partially explained by the fact that several tick selenoproteins have a signal peptide, targeting them for secretion. Since several selenogene transcripts (SelM, SelN, SelS, SelX) in addition to SOD3 (Figure 6) were downregulated in the *sef*-knockdown, this could have resulted in no detectable antioxidant activity in tick saliva (Figure 5). Moreover, many selenoproteins are predicted to be retained partially or exclusively within the endoplasmic reticulum. It has previously shown that oxidative stress targets the ER and secretory pathway [46]. If the secretory pathway was comprised, this could also have contributed to the loss in antioxidant activity in tick saliva (Figure 5). 

The critical importance of tick saliva to successful feeding and pathogen transmission cannot be understated. It is well accepted that the many tick-borne pathogens exploit the pharmacologically active molecules in tick saliva (saliva-activated transmission) [47]. Moreover, the role of tick selenoproteins, GPx and SelM, has been specifically shown to affect the acquisition of *B. burgdorferi* spirochetes and multiplication/colonization of *A. marginale*, respectively [15,16,32]. It is tempting to speculate that the role of SelM in the pathogen cycle of *A. mariginale* within *Dermacentor variabilis* is similar to the role of SelM in the pathogen cycle of *R. parkeri* within *A. maculatum*, but this does not appear to be the case and there are critical differences between these two studies that bear mentioning. First, SelM is overexpressed in response to *A. marginale* infection of *D. variabilis* [16], whereas SelM transcripts are much lower in *R. parkeri*-infected midguts (Figure 7). Second, microscopic work shows that the density of reticulated-form *A. marginale* was significantly higher in *D. variabilis* midguts of SelM knockdowns after acquisition/transmission feeding, whereas no *R. parkeri ompB* transcripts were detected in the midguts of the SEF knockdowns, though the overall pathogen burden within salivary gland and midgut tissues remained equivalent between control and the SEF knockdown (Figure 8). Third, Kocan et al. (2009) focused on one selenoprotein (SelM), while the knockdown SEF in *R. parkeri*-infected *A. maculatum* resulted in decreased transcripts of SelM and catalase, and increased transcripts of SelO, SelS, and SelT in tick midguts at various time points (Figure 7). The cellular role of SelO has not yet been determined, but there is some evidence to suggest it is an oxidoreductase-regulated protein kinase [34]. SelS resides within the ER membrane and is a cytosolic ATPase responsible for retrotranslocation of misfolded proteins from the ER [35]. The 2.5-fold increase in SelS transcriptional activity within *R. parkeri*-infected *A. maculatum* (Figure 7) suggests there is considerable stress to the ER. Furthermore, the decrease in transcription of catalase, which reduces the powerful oxidant H_2_O_2_, may further potentiate cellular oxidative stress. 

It is noted that geographically separated tick populations can contribute to difference in vector competency, which now best explains differences in regional patterns of disease transmission of *B. burgdorferi* [48,49]. It is possible that this could have contributed to the observed differences in selenoprotein transcriptional expression in pathogen-free and *R. parkeri*-infected *A. maculatum* midguts (Figure 7). However, an excellent paper has recently examined population dynamics of *A. maculatum*-ticks infected with *R. parkeri*. This work demonstrated that (1) 4 tick 16S haplotypes were not significantly different among regions of Mississippi, (2) there was no support for differentiation between northern and southern tick samples separated by more than 250 miles, and (3) no genetic variation was identified in any of the six selected gene targets of *R. parkeri* examined in the infected ticks, suggesting high levels of intermixing [50]. This suggests that the influence of potential tick population bottlenecks resulting from geographically distinct population would contribute relatively little to the overall differences between pathogen-free and *R. parkeri*-infected ticks. 

It is generally accepted that most tick bacterial pathogens follow similar growth and tick tissue migration patterns to *B. burgdorferi*. Spirochetes colonize the midgut and begin multiplying when feeding commences. Spirochetes then migrate within the hemolymph through the hemocoel, colonize the salivary glands, and are ultimately disseminated to the host through secretion [51]. Rickettsiae multiply in almost all tick organs, in particular the salivary glands and ovaries, and the migration of *R. felis* from midgut to salivary glands appears to be similarly dependent on blood meal feeding [52]. The presence of *R. parkeri* in salivary glands at 7 days post-infestation (Figure 8) indicates an unlikelihood that trafficking between the midguts and salivary glands was significantly inhibited by oxidative stress. 

The lack of antioxidant capacity in saliva (Figure 5) and the reduction in transcriptional activity of antioxidant genes in tick midguts SEF-knockdowns (Figure 6) indicates that both the salivary glands and midguts were affected by the SEF knockdown. It is conceivable that *R. parkeri* may have died within the midgut tissues as a result from ROS generated from digestion of the blood meal coupled with a failure in cellular antioxidant systems in SEF knockdowns. Oxidative stress, resulting from the degradation of hemoglobin, could result in oxidative killing of the microbial community in the tick midgut. This would explain the presence of heme-transporting lipoproteins, such as HeLp, in tick midguts [53]. Since the tick midgut harbors many beneficial endosymbionts, the presence of antioxidant factors, including selenoproteins, must be an adaptive mechanism to compensate for the deleterious effects of blood meal ingestion. This mechanism seems to be operative in protecting the *Aedes aegypti* gut microbial community from heme-induced toxicity [39]. However, if this was true one would expect *R. parkeri* would be similarly killed in salivary glands, when, in fact, the pathogen burden is twice as high. 

 A wide range of transcriptional changes occurred in the SEF knockdown, particularly in the hugely overrepresented class of secreted proteins and underrepresented oxidant metabolism/detoxification as well as many sequences with unknown functions (Table 3). This large shift in transcriptional activity could be, in part, accounted for by the 3,000-fold suppression of the SDS3 component of the Sin3 histone deacetylase corepressor complex in *R. parkeri*-infected *A. maculatum* (Figure 11). Sin3 regulates processes for organismal development and homeostasis, including mitochondrial biogenesis and cell death [54]. Sin3 recruits histone deacetylases to chromatin-bound transcription factors to repress the transcriptional expression of target genes through chromatin remodeling [45,55]. The CpG-binding protein MeCP2 can recruit the Sin3 complex, resulting in transcriptional repression via DNA methylation [56]. Given that the salivary proteins are products of multigenic families, the transcriptional expression of these gene families could be under the partial control of the Sin3 histone deacetylase corepressor complex. Surprisingly, a recent paper shows strong evidence that Sin3 acts predominantly as a transcriptional activator and regulates signaling of Jnk and Src pathway activity in a HDAC-independent manner [54]. The stress-activated protein kinase/Jun-amino terminal kinase (SAPK/JNK) is potently and preferentially activated by a variety of environmental stress including UV, ceramides, inflammatory cytokines, and in some instances growth factors and GPCR agonists [57]. The Src family of protein tyrosine kinases are important in the regulation of growth and differentiation [58]. It remains unclear if/how redox status influences the downregulation of components of the Sin3 complex as well as what other factors are in play. Intriguingly, selenites were shown to reactivate silenced genes in *Drosophila* [59], while naturally occurring alpha-keto acid metabolites of organoselenium compounds, including selenomethionine and Se-Methyl-L-selenocysteine were shown to inhibit histone deacetylase [60], and selenium organic compounds have been proposed to treat or prevent cancer by their histone deacetylase inhibitory activities [61]. Therefore, there is a possible cascade of events following SEF knockdown that in turn increases the availability of selenium-based metabolites that inhibit histone deacetylase with possible feedback suppression of the SIN3 corepressor complex transcript and overall change in transcript expression due to the epigenetic effects of histone acetylation. These hypotheses remain to be investigated. 

In conclusion, targeting SEF for RNAi did not affect feeding, vitellogenesis or fecundity, despite impacting total salivary antioxidant capacity. The global impact of RNAi silencing can be seen in lower transcriptional levels of SelM, SelN, SelS, SelX, SOD and, surprisingly, no concomitant increase in the transcriptional activity of other antioxidants studied. Additionally, no live *R. parkeri* was noted in the midguts of SEF knockdowns, and more than double the pathogen burden in salivary glands. The role of oxidative stress and the exact mechanism by which the SEF knockdown affects pathogen colonization, maintenance, trafficking, and proliferation remains to be precisely determined. While there is a mouse-model with susceptibility to *R. parkeri* infection [62], there are difficulties associated with this model including an inability to infest adult ticks on mice and the fact that mice are not the natural host for *R. parkeri*. The further development of an animal model of *R. parkeri* infection, perhaps using guinea pigs, could lead to a significant advancement in the understanding of the role of tick salivary factors in *R. parkeri* transmission and therefore generate potential targets for inhibiting disease transmission. Finally, our serendipitous RNA-seq finding of SEF knockdown leading to dramatic expression changes in transcripts coding for salivary secreted proteins in general, and on an overwhelming suppression of the SDS3 component of the Sin3 histone deacetylase corepressor complex points to an unexpected and unexplored link between SEF and epigenetics.

## Materials and Methods

### Ethics Statement

All animal experiments were performed in strict accordance with the recommendations in the Guide for the Care and Use of Laboratory Animals of the National Institutes of Health. The protocol of tick blood feeding on the sheep was approved by the Institutional Animal Care and Use Committee of the University of Southern Mississippi (protocol # 10042001). All efforts were made to minimize animal suffering.

### Ticks and animals

Pathogen-free *A. maculatum* were obtained from Oklahoma State University’s tick rearing center and were maintained at room temperature and 90% relative humidity under 14/10-hour light/dark photoperiod before infestation. *R. parkeri*-infected ticks were collected from the Mississippi Sandhill Crane National Wildlife Refuge (Gautier, MS) and infection was confirmed by QRT-PCR. *A. maculatum* were fed on sheep and were either allowed to feed to repletion or removed between 18 hrs-216 hrs, depending on the experimental protocol. Adult ticks were fed specifically for this study and all animal studies were performed in accordance with protocols approved by the Institutional Animal Care and Use Committee (IACUC) at the University of Southern Mississippi.

### Bioinformatics analyses

The coding sequence from *A. maculatum* SEF (GenBank ID: KC989559) was obtained by pyrosequencing an *A. maculatum* salivary gland cDNA library [22]. Nucleotide sequences were conceptually translated, initially aligned using ClustalX2 [63], refined by eye, and graphically presented using Jalview 2.7 [64,65]. Phylogenetic relationships were inferred using MEGA 5 using the maximum parsimony method [66]. 

### Tick dissection and saliva collection

Tick tissues were dissected in ice-cold M-199 buffer [67,68]. After removal, salivary glands and midguts were washed gently in the same ice-cold buffer. Tissues were stored at -80°C in RNAlater (Invitrogen) for transcriptional studies or in 0.5M PIPES, pH 6.8, containing 20 mM EGTA, protease inhibitor cocktail (Amresco) and 40% glycerol for protein analyses. Tick saliva was collected as previously described [69]. Dopamine and theophylline (1 mM each) in 20 mM MOPS buffered saline with 3% DMSO, pH 7.0 were injected as a stimulant for salivation [70]. The saliva was used immediately after collection or stored at -80°C.

### RNA preparation and cDNA synthesis

Total RNA was isolated from salivary glands and midguts dissected from unfed and partially-fed adult female ticks using illustra RNAspin Mini RNA isolation kit (GE Healthcare). The concentration of total RNA was determined spectrophotometrically and aliquots were stored at -80°C. Total RNA was reverse-transcribed using MMLV reverse transcriptase according to the manufacturers’ instructions. 

### Quantitation of selenoprotein mRNA

Quantitative real-time PCR (QRT-PCR) was performed on a Bio-Rad C1000 Thermocycler fitted with the CFX96 Realtime System for fluorescence detection. SYBR Green PCR Mix was obtained from Fermentas and the manufacturer’s instructions were followed. Primer sequences used for QRT-PCR are listed in Table 2. Reactions (25 μl) containing 500 ng of *A. maculatum* salivary gland or midgut cDNA were run under the following PCR protocol: 50°C for 3 minutes, 95°C for 10 minutes, followed by 35 cycles of 95°C for 15 seconds, 60°C for 30 seconds, 72°C for 30 seconds. The fluorescence was read after the final extension step. Samples were run in triplicate with no-RT and no-template controls. Expression of each selenogene and the reference gene, actin, was used to calculate expression values using the Bio-Rad data analysis software package [71]. These values were then normalized to levels observed in unfed ticks for ease in comparison. A two-fold change in expression was the criteria for statistical significance in QRT-PCR assays and was determined using the Bio-Rad CFX manager (ver. 6).

### Synthesis of sef-dsRNA and microinjection

The SEF PCR product was joined to the T7 promoter linker using the Block-iT T7 TOPO kit (Invitrogen). The TOPO linking reaction was used as a template for a PCR reaction containing the T7 PCR primer and a *sef* primer to produce sense and antisense linear DNA template. After transcription, the sense and antisense ssRNA was purified, annealed, and verified in size by agarose gel electrophoresis. To investigate the role of selenoproteins in tick feeding success *in vivo*, 50 unfed female ticks were injected with 1000 ng of *sef-*dsRNA into the hemocoel using a Hamilton syringe fitted with a 33-gauge needle. Control ticks were injected with 1000 ng LacZ-dsRNA. After injection of dsRNA, ticks were kept at 37°C overnight under high humidity to confirm tick survival, and then infested on a sheep [72]. 

### RNA interference phenotype

Ticks injected with dsRNA were allowed to feed for nine days and were then removed and weighed on an analytical balance. Some of the ticks injected with dsRNA were allowed to feed to repletion and the size of the egg mass and hatching was monitored. 

### Total Antioxidant Capacity

The total antioxidant capacity of pooled tick saliva was determined according to the manufacturer’s protocol using QuantiChrome Antioxidant Assay Kit (Bioassay Systems). Trolox was used as the antioxidant standard. 

### Quantification of *R. parkeri* in *A. maculatum* tissues

*A. maculatum* ticks were dissected and the salivary glands and midguts were placed in RNAlater (Invitrogen) and stored at -80°C until RNA could be extracted from these tissues. DNA was extracted from the remaining carcass (dissected tick without the salivary glands and midgut) and used as a template for a real-time PCR assay designed for specific detection of the *R. parkeri ompB* gene using gene-specific primers (Rpa129F/Rpa224R) and Rpa188 as a probe (Table 2) and has been previously described [73,74]. Real-time PCR was performed using a Bio-Rad C1000 Thermocycler fitted with the CFX96 Realtime System for fluorescence detection. After initially identifying which ticks tested positive for the *R. parkeri*, cDNA was prepared from the midguts and salivary glands from three ticks. Two independent QRT-PCR runs were performed based on these samples. 

### RNA-seq analysis

Pooled total salivary gland RNA was used to construct six libraries from either uninfected or *R. parkeri*-infected *A. maculatum* at both 3 and 5 days post-infestation, as well as from *R. parkeri*-infected ticks injected with AmSEF- or LacZ-dsRNA collected after seven days of feeding. Library construction and RNA-seq assays were performed by Otogenetics (Norcross, GA). Briefly, the integrity and purity of total RNA were assessed using the Agilent Bioanalyzer and OD_260/280_. Five micrograms of total RNA was treated using the Ambion GLOBINclear-Human kit to deplete globin mRNA resulting from any recent blood meal, and subsequently 1-2 µg of cDNA was generated from the depleted sample using the Clontech SmartPCR cDNA kit from 100 ng of total RNA. Adaptor sequences were removed by restriction digestion, and the resulting cDNA was fragmented using Covaris M220 focused-ultrasonicator (Woburn, MA) and subjected to Illumina library preparation using NEBNext reagents. The quality, quantity, and size distribution of Illumina libraries were determined using the Agilent Bioanalyzer 2100. The libraries were then submitted for Illumina HiSeq2000 sequencing according to the standard operation. Paired-end 90-100 nucleotide reads were generated and checked for data quality using FASTQC (Babraham Institute, Cambridge, UK). The RNA-seq reads were then mapped to the *A. maculatum* sialotranscriptome [22] using a data analysis pipeline developed by José M. C. Ribeiro. Briefly, Illumina reads were blasted (blastn) against the *A. maculatum* coding sequences with a word size of 25 and allowing up to 5 matches if these had the same score, maximum of 1 gap and minimum identity of 98%. The data was mapped to a hyperlinked Excel spreadsheet (file S1) available from http://exon.niaid.nih.gov/transcriptome/Amb_maculatum/Sef/Ambmac-Sefi-web.xlsx. 

Raw reads were deposited in the Sequence Read Archive of the National Center for Biotechnology information under Bioproject PRJNA217443, Biosample SAMN02338764 and runs SRR959015 (Sef) and SRR959016 (LacZ).

### Statistics

All data are expressed as means ± SEM. Statistical significance was determined by Student’s *t*-test; differences among multiple experimental groups was determined by ANOVA (SigmaPlot ver. 11). A two-fold change in gene expression with p<0.05 was considered statistically significant.

## Supporting Information

File S1 RNA-seq data analysis (XLSX)
